# New ternary powder metallurgy Ti alloys via eutectoid and isomorphous beta stabilisers additions

**DOI:** 10.1038/s41598-023-28010-7

**Published:** 2023-01-20

**Authors:** M. Paul, Y. Alshammari, F. Yang, L. Bolzoni

**Affiliations:** 1 School of Engineering, The University of Waikato, Hamilton, 3240 New Zealand; 2 College of Engineering, International University of Science and Technology in Kuwait, Mohamad Bin Qasim Street, Ardiya, Kuwait

**Keywords:** Structural materials, Mechanical engineering

## Abstract

A group of new ternary Ti alloys bearing eutectoid and isomorphous beta stabilising elements was created to be manufactured through the conventional powder metallurgy route. The effect of the simultaneous addition of the same amount of Mn and Nb on the manufacturability, properties, and hardening behaviour was investigated. The ternary alloys are composed of the α-Ti and β-Ti phases and have a lamellar microstructure resulting from the slow cooling upon sintering. However, the size of the equiaxed α grains and of the α + β lamellae is monotonically reduced, especially the interlamellar spacing, as the amount of alloying elements increases. Due to their physical properties, Mn enhances and Nb hinders densification during sintering resulting in a decreasing trend of the relative density with the alloying elements content. Consequently, the resistance to plastic deformation increases (UTS, 514–726 MPa), the ductility decreases (elongation, 13.2–2.6%), and the fracture mode changes from intergranular to transgranular. The new ternary alloys share the same hardening mechanism, but the amount of deformation after necking is, generally, higher for lower amounts of Mn and Nb.

## Introduction

Titanium alloys have been widely applied in the biomedical sector as orthopaedic and dental materials due to their high strength, comparatively low Young modulus, good corrosion resistance, and bioinert behaviour^1–5^. However, currently used Ti-based alloys generally relies on expensive and critical alloying elements, their stiffness is still higher than that of human bones, and some of the elements have neurotoxic and cytotoxicity issues^6,7^.

Amongst the alloying elements of titanium alloys, manganese (Mn) is one of the strongest stabilisers of the allotropic β titanium phase^8^, and can also reduce the cost of the alloy^9,10^. From a biological standpoint, Mn plays a key role in the osteogenesis and in the mechanism of bone resorption^11,12^, where Mn deficiencies have been reported to lead to disproportionate growth of the skeleton^11^. Other human body and brain functions are also influenced by trace Mn^13^. Although dietary intake of Mn is essential and its cytotoxicity is remarkably lower than that of V^14^, limiting the amount of Mn to less than 8 wt.% in Ti alloys prevents intoxication risks and the formation of the brittle metastable ω phase^15^.

The fabrication of binary Ti–Mn alloys via casting has been analysed extensively^8,10–12,16^; for example, Kim et al.^16^ produced Ti–xMn alloys (x = 5, 10, 15, and 20 wt.%) were the samples were remelted seven times and heat treated using a tube furnace under argon atmosphere for 4 h, at a temperature lower than the respective solidus temperatures (150 °C), before cooling in the furnace at 10 °C/min down to 600 °C and final air cooling. With the addition of Mn the proportion of the equilibrium α phase decreases and the amount of stabilised β phase increases, leading to higher hardness and better oxidation protection ability.

Development of binary Ti–xMn alloys (x = 8, 9, 12, 13, 15, and 17 wt.%) via powder metallurgy using metal injection moulding has been considered by Fernandes Santos et al.^15^ using gas atomised Ti and fine Mn powders with a particle size lower than 45 μm. Rectangular samples were obtained through injection moulding of blends of powders mixed with an organic binder and sintered under vacuum at 1100 °C for 8 h prior to their final solution treatment at 900 °C for 1 h. With the Mn content analysed, the alloys were characterised by β phase grains, but the presence of the brittle metastable ω phase was also detected for alloys with 8 ≤ Mn ≤ 13 wt.%. Consequently, the hardness increased but the yield stress (YS) and ultimate tensile strength (UTS) as well as the ductility decreased with the increment of the Mn addition.

The incorporation of Nb in titanium alloys has been widely considered as Nb is a isomorphous β stabiliser which is beneficial for lowering the Young’s modulus of the alloy and is less cytotoxic/non-toxic compared to other elements^17^. Thus, binary Ti–Nb alloys have attracted great attention in biomedical devices such as dental implants due to their superior biocompatibility, corrosion resistance as well as mechanical properties^18,19^. A lower Nb concentration in Ti–Nb alloys is reported to induce the shape memory effect, which is employed in dentistry for orthodontic wires^20^. Nb is a rare expensive metal with a very high melting point, thus it is best to design Ti–Nb alloys with a low Nb concentration to benefit cost savings and reduce manufacturing steps (i.e. to avoid operating at very high temperatures)^21^.

The fabrication of binary Ti–Nb alloys via casting has been extensively studied^17–21^; for example Zhang et al.^21^ analysed the mechanical and biological properties of Ti–xNb alloys (x = 5, 10, 15, 20, and 25 wt.%) by means of a vacuum consumable-electrode arc furnace and subjected to a series of forging-cogging, rolling, straightening and scaling operations. All the Ti–xNb alloys were characterised by the presence of both the α and β phases, where the amount of β phase increased and that of the α phase decreased as the amount of Nb added was increased. Consequently, the strength (both YS and UTS) increased and the ductility decreased with higher Nb additions. The Ti–xNb alloys demonstrated favourable biocompatibility and a promising anti-corrosion capacity from the measurement of the concentration of released metallic ions, morphology and chemical composition.

Although to a lesser extent, as the amount of Nb added was always higher than 10 wt.%^22–24^, the fabrication of binary Ti–Nb alloys via powder metallurgy has also been considered; for example, Zhao et al.^22^ used the metal injection moulding powder metallurgy route to produce the Ti–xNb alloys (x = 10, 16, and 22 wt.%) starting from Ti (< 45 μm) and Nb (< 110 μm) elemental powders. The increase of Nb content led to an increase of porosity in the as-sintered samples due to the higher ratio of coarse Nb powder used and the slow diffusion of Nb, both decelerating the sintering process resulting in higher porosity. Therefore, it was found that the addition of Nb lowered the Young's modulus and the elongation but increased the strength. The as-sintered Ti–Nb alloys mainly consisted of α + β Widmanstätten structures with similar grain sizes of about 100–300 μm.

Regarding the development of Ti alloys where Mn and Nb were simultaneously added, the work performed is significantly lower and the main two investigations available in literature, to the best knowledge of the authors, are the work of Chen et al.^25^ via casting and that of Ehtemam-Haghighi et al.^26^ via powder metallurgy. Specifically, Chen et al.^25^ prepared a series of Ti–27Nb–xMn alloys (x ~ 0, 1, 3, 5, 7, and 9 wt.%) by means of cold crucible levitation melting in inert argon atmosphere. The alloys were subsequently cold rolled into a plate with a thickness reduction of ~ 60% and further solution treated at 900 °C for 30 min prior to water quenching. The characterisation of the cast alloys indicated that the α'' and β phases in the Ti–27Nb alloy were modified by the addition of Mn, resulting in the formation of a single β phase. The tensile strength of the Ti–27Nb–xMn alloys initially increased, reaching its maximum for the Ti–27Nb–5Mn alloy, and then decreased due to the presence of the athermal ω phase, the β phase, and the solid-solution strengthening effect of Mn. Ehtemam-Haghighi et al.^26^ used the conventional press and sinter powder metallurgy processing from blended elemental powders (Ti < 50 μm, Mn < 10 μm, and Nb < 50 μm) to manufacture Ti–7Mn–xNb alloys (x = 3, 7, and 10 wt.%), which were then heated up to 1170 °C, held isothermally for 8 h before furnace cooling at the rate of 4 °C/min. The increment of the amount of Nb added led to enhancement of the stabilisation of the β phase, whose relative amount therefore increased, even though all the alloys were characterised by a lamellar microstructure. Consequently, the compression strength and hardness decreased, and the elongation slightly increased, with the Nb content.

From literature, binary Ti–Mn and Ti–Nb alloys have been well studied but the potential of the ternary Ti–Mn–Nb alloying system has not been fully exploited as the few works available^25,26^ did not characterised the alloy in the as-produced state, as the Ti–Mn–Nb alloys were always heat treated, if not also plastically deformed via cold rolling. Moreover, the study of Chen et al.^25^ considered very high amount of Nb (i.e. 27 wt.%) whereas the study of Ehtemam-Haghighi et al.^26^ had a fairly high amount of Mn (i.e. 7 wt.%) and both studies had the content of one of the two alloying elements fixed. This means that, to the best knowledge of the authors, there are no studies on the development of ternary Ti–Mn–Nb alloys with relatively low additions of Mn and Nb aiming to produce α + β and no studies were the amount of added Mn and Nb is changed simultaneously. Additionally, no tensile properties of ternary Ti–Mn–Nb alloys produced via powder metallurgy have been reported. To fill the gap in the current literature, the aim of this study is to analyse the development of ternary Ti–xMn–xNb alloys (x = 0.5, 1, 3.5, and 5 wt.%) and characterise the properties achievable in the as-produced state, when these alloys are manufactured via powder metallurgy. The Ti–xMn–xNb alloys were, therefore, characterised in terms of relative density/porosity, microstructural analysis (optical and electron microscopy as well as XRD), and mechanical properties (tensile behaviour and hardness).

## Results

**Figure 1:**
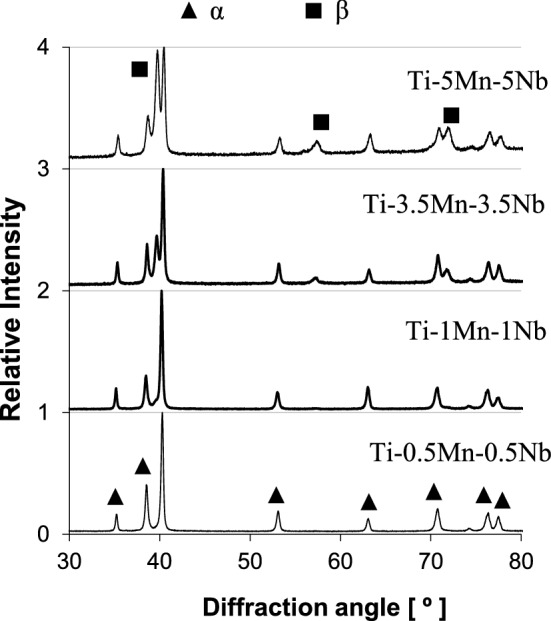
XRD spectra of the Ti–xMn–xNb alloys.

Figure 1 shows the results of the XRD analysis performed on the Ti–xMn–xNb alloys where it can be seen that the α-Ti phase is the predominant phase composing the materials regardless of their actual chemistry. The β-Ti phase is detected exclusively in the Ti–3.5Mn–3.5Nb and Ti–5Mn–5Nb alloys, and in the latter the relative intensity of the strongest diffraction peak of the (110) family of planes of the β-Ti phase is almost as high as that of the diffraction peak of the (101) plane of the α-Ti phase.

**Figure 2:**
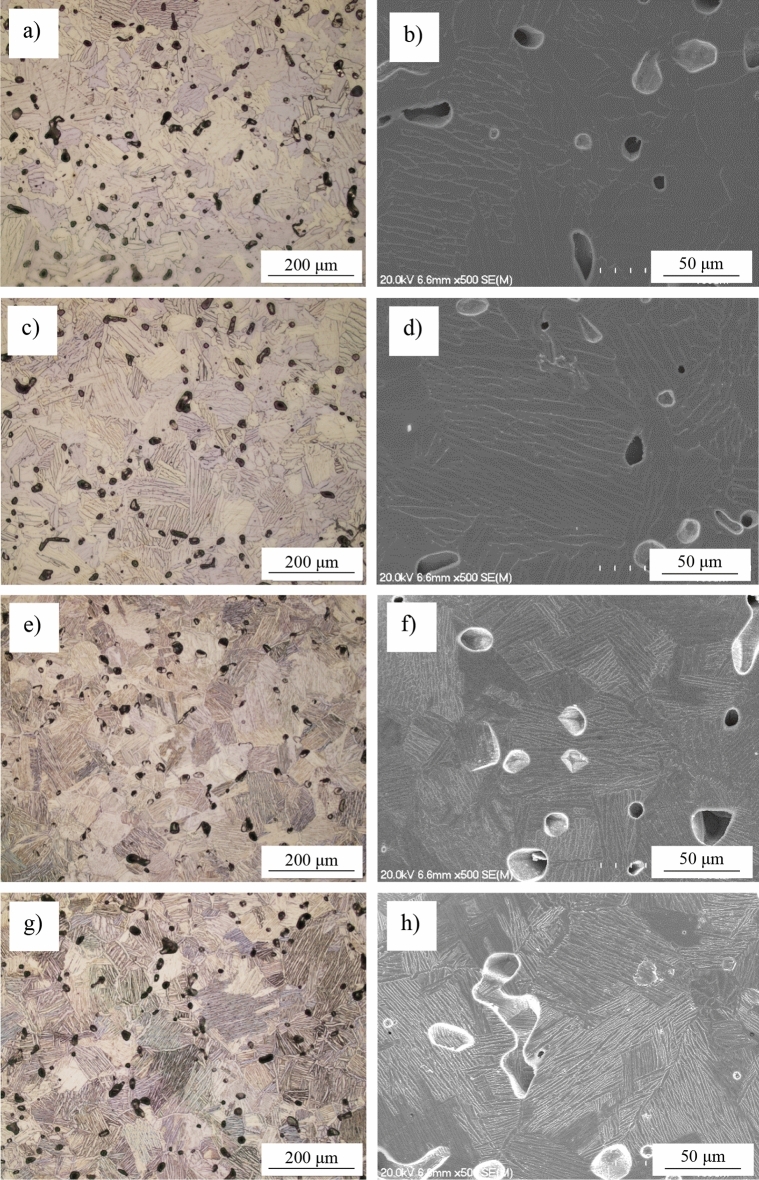
Optical and SEM micrographs, respectively, of the Ti–xMn–xNb alloys: (**a**,**b**) Ti–0.5Mn–0.5Nb, (**c**,**d**) Ti–1Mn–1Nb, (**e**,**f**) Ti–3.5Mn–3.5Nb, and (**g**,**h**) Ti–5Mn–5Nb.

Figure 2 shows the results of the microstructural analysis performed on the Ti–xMn–xNb alloys. Regardless of the chemical composition, a small amount of residual porosity is present in the microstructure, which is typical for powder metallurgy Ti alloys^27,28^. The majority of the pores are isolated, they have either spherical or elongated morphology, they are primarily located at the boundaries between phases, and their volumetric fraction slightly increases with the amount of alloying elements added. In terms of microconstituents, the microstructure of the Ti–0.5Mn–0.5Nb alloy is similar to that of the Ti–1Mn–1Nb alloy, and that of the Ti–3.5Mn–3.5Nb alloy to that of the Ti–5Mn–5Nb alloy, even though all of them have a lamellar structure typical of α + β Ti alloys^29,30^. In the former case, when the combined addition of Mn and Nb is ≤ 2 wt.%, the Ti–xMn–xNb alloys have a coarse lamellar structure composed of equiaxed α-Ti grains boundaries and α + β lamellae. In the latter case, when the combined addition of Mn and Nb is ≥ 7 wt.%, the Ti–xMn–xNb alloys have a fine lamellar structure composed of equiaxed α-Ti grains boundaries and α + β lamellae. The incremental combined addition of Mn and Nb progressively reduces the size of the equiaxed α-Ti grains, increases the amount of stabilised β-Ti phase, and significantly refines the lamellar spacing of the α + β lamellae.

**Figure 3:**
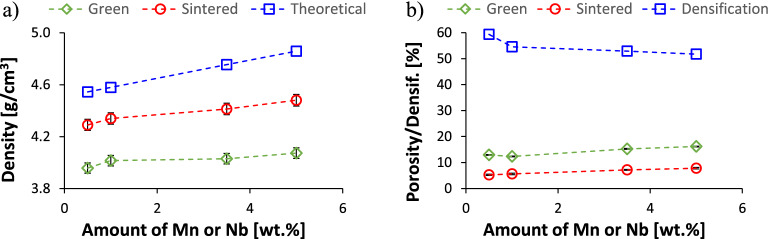
Density (**a**) and porosity/densification (**b**) of the Ti–xMn–xNb alloys.

Figure 3 shows the variation of the density as well as porosity/densification of the Ti–xMn–xNb alloys. The density of the as-pressed samples (i.e. green, 3.96→4.07 g/cm^3^), that of the sintered specimens (4.29→4.48 g/cm^3^), and the theoretical density of the alloys (4.54→4.86 g/cm^3^), all increase linearly with the content of Mn and Nb as both these alloying elements have higher density than Ti. The amount of porosity left by either the pressing (12.9→16.2%) or sintering (5.2→7.8%) of the Ti–xMn–xNb powders blends also increases linearly with the amount of alloying elements added, whereas the densification parameter decreases from 59.3% to 51.8%.

**Figure 4:**
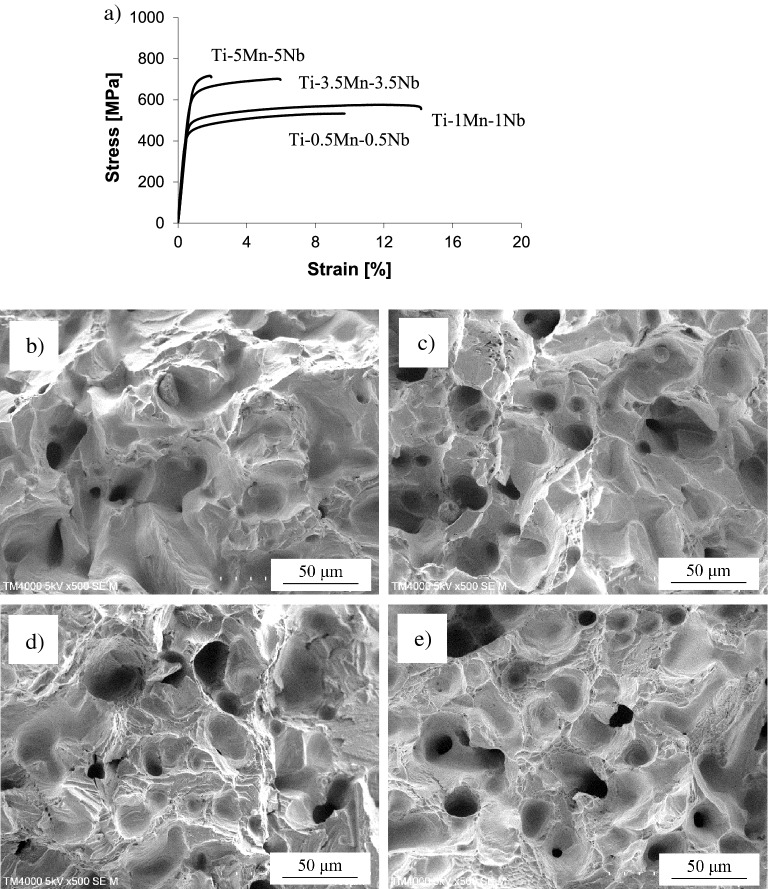
Representative stress/strain curves (**a**) and micrographs of the fracture surface of the Ti–xMn–xNb alloys: (**b**) Ti–0.5Mn–0.5Nb, (**c**) Ti–1Mn–1Nb, (**d**) Ti–3.5Mn–0.3Nb, and (**e**) Ti–5Mn–5Nb.

Figure 4 shows representative stress/strain curves as well as typical results of the fractographic analysis of the Ti–xMn–xNb alloys where it can be seen that, regardless of the chemistry of the alloy, both elastic and plastic deformation happen in the Ti–xMn–xNb alloys, but the actual strength and ductility strongly depends on the composition. More in detail, the strength/ductility pair of the Ti–0.5Mn–0.5Nb alloy is more comparable to that of the Ti–1Mn–1Nb alloy, and that of the Ti–3.5Mn–3.5Nb alloy to that of the Ti–5Mn–5Nb alloy. Consequently, the failure mode detected in the fracture surface of the tensile samples also changes depending on if the combined addition of Mn and Nb is ≤ 2 wt.% or ≥ 7 wt.%. On the one side, for lean alloyed Ti–xMn–xNb alloys, the material prevalently fails in a ductile manner due to the intergranularly plastic deformation of the coarse α-Ti lamellae and the residual pores do not seem to play a significant role, as they remain mainly undeformed. On the other side, for more heavily alloyed Ti–xMn–xNb alloys, the failure of the material is more brittle in nature with combined intergranular and transgranular failed zones and greater influence from the residual porosity. In particular, either zones with highly deformed pores or cracks initiated at the pores are present.

**Figure 5:**
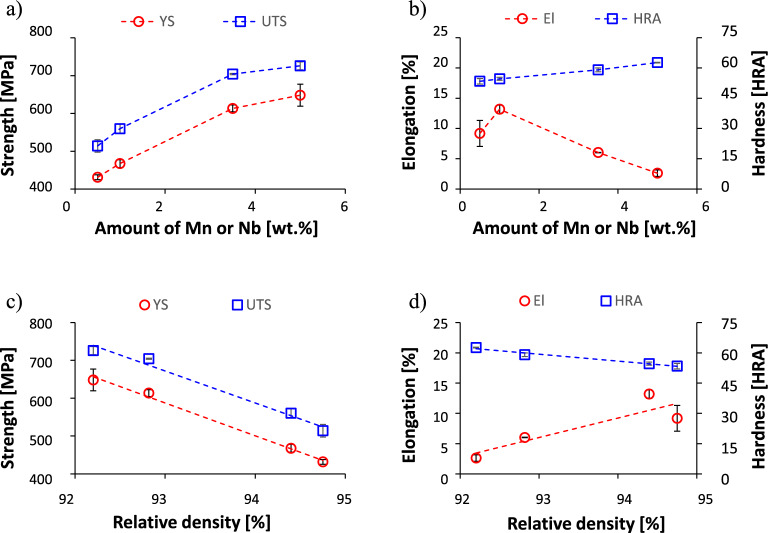
Average mechanical properties of the Ti–xMn–xNb alloys: (**a**) yield stress and ultimate tensile strength vs amount of Mn or Nb, (**b**) elongation and hardness vs amount of Mn or Nb, (**c**) yield and ultimate tensile strength vs relative density, (**d**) elongation/hardness vs relative density.

Figure 5 shows the average mechanical properties of the Ti–xMn–xNb alloys either versus the amount of alloying elements or versus the relative density of the sintered samples. In agreement with the representative stress/strain curves, the resistance to plastic deformation increases with the incremental addition of Mn and Nb as YS ranges from 431 ± 6 to 648 ± 29 MPa and UTS ranges from 514 ± 17 to 726 ± 11 MPa. The hardness also linearly increases with the content of Mn and Nb and ranges from 53 ± 1.4 HRA to 63 ± 0.2 HRA. In the case of the elongation, it can be seen that the ductility of the Ti–xMn–xNb alloys initially increases with the addition of Mn and Nb reaching the maximum value of 13.2 ± 0.7% for the Ti–1Mn–1Nb alloy and, afterwards, decreases to the lowest value of 2.6 ± 0.5% for the Ti–5Mn–5Nb alloy. When analysed against the relative density, in order to identify the power of the contribution of the residual pores, both the strength (i.e. YS and UTS) and hardness decrease with the increment of the relative density, indicating that residual porosity is not their main controlling aspect. Nonetheless, in the case of the ductility, an increasing linear relationship between the elongation and the relative density is found, meaning that the residual pores have a much greater impact on the ductility^31,32^ than on the strength of the Ti–xMn–xNb alloys.

**Figure 6:**
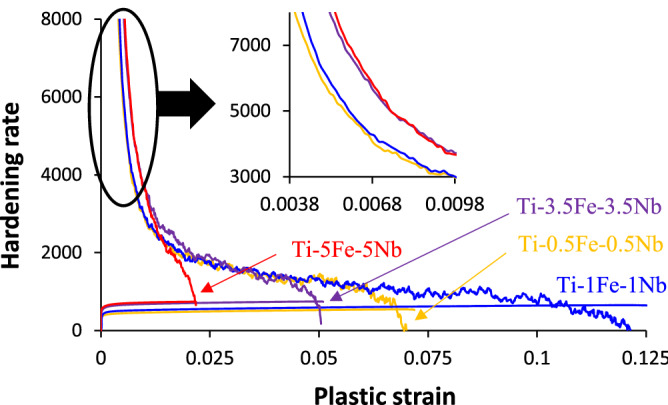
Hardening rate of the Ti–xMn–xNb alloys.

Figure 6 shows the hardening rate of the Ti–xMn–xNb alloys calculated as the ratio between the derivatives of the true stress and true plastic strain from the engineering stress/strain curves. The hardening rate curves of the Ti–xMn–xNb alloys have similar shape, indicting a similar deformation mechanism, where at low true plastic strain values the hardening rate is high and decreases significantly for small plastic strain increments for all the Ti–xMn–xNb alloys. Nevertheless, it can be noticed that, once again, the hardening behaviour of the Ti–0.5Mn–0.5Nb alloy coincides with that of the Ti–1Mn–1Nb alloy, and that of the Ti–3.5Mn–3.5Nb alloy with that of the Ti–5Mn–5Nb alloy (see inset), reflecting the impact of the actual chemistry of the alloy. At plastic strains ≥ 0.01, the alloys enter the last strain hardening stage, which is controlled by the equilibrium between formation and annihilation of dislocations^33^. This strain hardening regime is in the range of E/50 and, therefore, it is directly dependent on the chemistry of the alloy where, generally, the higher the amount of Mn and Nb, the higher the rate at which the strain hardening rate decreases. This results in the decrease of the uniform ductility for higher contents of Mn and Nb. Moreover, attending to the Considère criterion^34^, the amount of deformation after necking, which is identified by the intersection of the hardening rate curve with the true stress/true strain curves shown in Fig. 6, is higher for lower amount of Mn and Nb. The only exception is the Ti–1Mn–1Nb alloy, which has the highest uniform ductility and ability to withstand plastic deformation after necking has begun.

## Discussion

New Ti–xMn–xNb alloys were designed on the basis of the simultaneous addition of the same content of Mn and Nb where the former is a eutectoid and the latter an isomorphous stabiliser of the β-Ti phase. The new Ti–xMn–xNb alloys were designed to be manufactured via the blended elemental powder metallurgy approach has it offers to possibility to easily tailor the chemistry of the alloy. For that, elemental powders of the desired chemical elements were mixed in the right ratio, which leads to proportional increment of the density of the alloy (Fig. 3a) as both Mn and Nb have higher density than Ti. The Ti–xMn–xNb alloys powder blends were pressed into shaped samples by means of cold uniaxial pressing finding that the density of the alloys increases with the incremental content of Mn and Nb but the amount of residual porosity present in the pressed samples (i.e. green porosity in Fig. 3b) also increases. This means that the incremental addition of the Mn and Nb powders to the Ti powder decreases the compressibility of the powder blends, despite the fact that the three powders have particle morphology ideal (irregular and angular) for cold uniaxial pressing. The decrement of the compressibility is, therefore, due to the combination of particle size misfit, as both the Mn and Nb powders used have lower maximum particle size than Ti, and the difference in terms of deformability of the individual powders, as both the Mn and Nb are harder than Ti.

Sintering of the pressed Ti–xMn–xNb alloys samples does not change the trends found in the pressed samples as both the density and the residual porosity left during the sintering stage increase with the amount of Mn and Nb. The amount of residual porosity is influenced by both the compressibility of the powder blends and the dissolution of the Mn and Nb powder particles. Figure 3b) shows that the gap between the porosity found in the green and sintered samples is almost constant meanwhile the densification of the alloy decreases with the incremental addition of Mn and Nb. As the Ti–xMn–xNb alloys samples were all sintered under the same conditions (i.e. 1300 °C during 2 h), the decrement of the densification and the final amount of residual porosity are, thus, dictated by the diffusion of the alloying elements to homogenise the chemistry of the alloy. Mn has lower melting point than Ti and good diffusivity at the sintering temperature used, whereas Nb has much higher melting point than Ti and poor diffusivity in Ti at 1300 °C. Thus, the relative density values achieved (92.2→94.8%) are the compromised between Mn promoting and Nb hindering the densification of the Ti–xMn–xNb alloys. It is worth noticing that, however, the obtained relative density values are common for powder metallurgy Ti alloys^15,22,27,28^ where the achievement of a minimum of 92% of relative density generally indicates having reached the last stage of sintering. This is confirmed by the microstructural analysis (Fig. 2) of the Ti–xMn–xNb alloys where isolated primarily spherical residual pores were identified, which are typical of the last stage of sintering, where formation of isolated spherical pores by coalescence and coarsening of the microstructural features occur.

Regardless of the actual chemistry of the material, the Ti–xMn–xNb alloys are characterised by a lamellar structure composed of equiaxed α-Ti grains boundaries and α + β lamellae, where the α-Ti and β-Ti phases are the only phases detected during XRD analysis (Fig. 1). A lamellar structure is typical of α + β Ti alloys^29,30^ and it is formed upon the slow cooling of the alloy through its β transus temperature, where the equiaxed prior β-Ti grains transform into equiaxed α-Ti grains boundaries. Parallel alternating lamellae of the α-Ti and β-Ti phases nucleate and grow within the equiaxed α-Ti grains following one of the twelve specific orientation relationships between the body centred cubic lattice of β-Ti and the hexagonal closed packed lattice of α-Ti. The actual features of the lamellar structure of the Ti–xMn–xNb alloys are, however, highly influenced by the total amount of alloying elements added. Specifically, the Ti–0.5Mn–0.5Nb and Ti–1Mn–1Nb alloys have a coarse lamellar structure composed of equiaxed α-Ti grains, whose size is, respectively, comparable or bigger to that of the particle size of the starting powders, and coarse lamellae whose interlamellar spacing is slightly smaller in the case of the Ti–1Mn–1Nb alloy. The Ti–3.5Mn–3.5Nb and Ti–5Mn–5Nb alloys are characterised by a much finer lamellar structure composed of equiaxed α-Ti grains, whose size is smaller with respect to the starting powders, and α + β lamellae whose size is significantly refined by the higher addition of Mn and Nb. From the combined results of the XRD and microstructural analysis, it can thus be concluded that the Ti–0.5Mn–0.5Nb and Ti–1Mn–1Nb alloys are actually near-α alloys due to their slow amount of β-Ti phase stabilised, whilst the Ti–3.5Mn–3.5Nb and Ti–5Mn–5Nb alloys are α + β alloys.

The Ti–xMn–xNb alloys show an easlitc + plastc deformation behaviour when subjected to a uniaxial tensile load (Fig. 4) where the transition from one to the other and the actual extent of the plastic deformation is dictated by the microstructural features. Specifically, the phases present in the microstructure and their features have a stronger effect on the mechanical behaviour of the Ti–xMn–xNb alloys than the amount of residual porosity (Fig. 5c); even though the latter has a stronger influence on the ductility (Fig. 5d). Because of the specific amount of alloying elements added, the stress/strain curves (Fig. 4) and average mechanical properties (Fig. 5) of the Ti–0.5Mn–0.5Nb and Ti–1Mn–1Nb alloys are close together and so are those of the Ti–3.5Mn–3.5Nb and Ti–5Mn–5Nb alloys. Such behaviour is related to the formation of a coarse and fine lamellar microstructure, respectively, for the Ti–0.5Mn–0.5Nb and Ti–1Mn–1Nb alloys, and for the Ti–3.5Mn–3.5Nb and Ti–5Mn–5Nb alloys. The increment of the amount of stabilised β-Ti phase leads to the progressive refinement of the size of the equiaxed α-Ti grains and a significantly refinement of the lamellar spacing of the α + β lamellae. This justifies the increase in strength and hardness and the decrease in ductility of the Ti–xMn–xNb alloys with the amount of alloying elements. Because the Ti–xMn–xNb alloys of this study share the same lamellar structure, their hardening rate curves have similar shape (Fig. 6), but the actual hardening behaviour is alloy-specific. In the last strain hardening stage, higher strain hardening decreasing rates are obtained for higher amounts of alloying elements, meaning that dislocations formation overpowers dislocations annihilation^33^, leading to lower values of uniform ductility and deformation after necking. Accordingly, on the one side, the Ti–0.5Mn–0.5Nb and Ti–1Mn–1Nb alloys fail in a ductile manner through the transgranular fracture of the coarse α-Ti lamellae without much contribution from the residual porosity. On the other side, the Ti–3.5Mn–3.5Nb and Ti–5Mn–5Nb alloys fail in a more brittle manner where both intergranular and transgranular fracture areas are present, and the residual porosity contributes to the failure mode.

**Figure 7:**
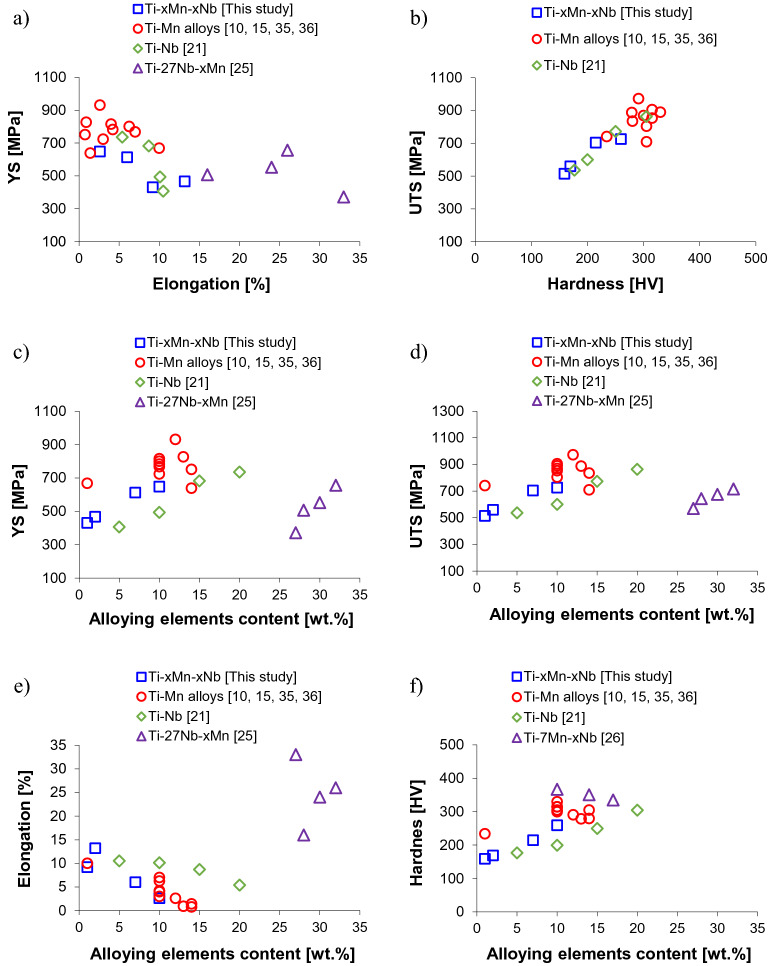
Comparison of the mechanical properties of the Ti–xMn–xNb alloys with current literature on binary Ti–Mn, binary Ti–Nb and ternary Ti–Mn–Nb alloys^10,15,21,25,35,36^: (**a**) YS vs elongation, (**b**) UTS vs hardness, (**c**) YS vs amount of alloying elements, (**d**) UTS vs amount of alloying elements, (**e**) elongation vs amount of alloying elements, and (**f**) hardness vs amount of alloying elements.

Figure 7 shows the comparison of the tensile behaviour of the Ti–xMn–xNb alloys of this study with current literature on binary Ti–Mn, binary Ti–Nb, and ternary Ti–27Nb–xMn alloys (x ~ 0, 1, 3, 5, 7, and 9 wt.%)^10,15,21,25,35,36^. With reference to the latter, it is worth noticing that Ehtemam-Haghighi et al.^26^ measured compressive properties, and thus could not be included in the comparison, whilst Chen et al.^25^ used casting followed by cold rolling, solution treatment and final quenching from 900ºC and reported tensile properties but did not quantify the hardness of the Ti–27Nb–xMn alloys. In terms of YS/elongation pairs (Fig. 7a), the Ti–xMn–xNb alloys of this study have comparable properties to some of the binary Ti–Mn and Ti–Nb alloys whilst the Ti–27Nb–xMn alloys have higher ductility for similar YS values. The differences are mainly due to the actual composition of the alloys and the way they were manufactured and, therefore, a decreasing trend can be seen between the YS and the elongation. Specifically, the binary Ti–Nb and Ti–27Nb–xMn alloys were thermomechanically processed, and so some of the binary Ti–Mn alloys, meaning that they have no residual porosity, where the residual pores greatly affect the ductility. With respect to the UTS/hardness pairs (Fig. 7b), those of the Ti–xMn–xNb alloys of this study are similar to those of binary Ti–Nb alloys and lower than those of binary Ti–Mn alloys, where a monotonically increasing trend is found between UTS and hardness.

A better understanding of the actual relative performance of the Ti–xMn–xNb alloys of this study with respect to other Mn- and Nb-bearing Ti alloys is achieved considering the variation of each individual property as a function of the total amount of β stabilising alloying elements. From Fig. 7c), the Ti–xMn–xNb alloys have higher and lower YS, respectively, compared to Ti–Nb and Ti–Mn alloys for the same amount of β stabilisers, highlighting the different strengthening effect that Mn as eutectoid and Nb as isomorphous elements have on Ti. The YS of the Ti–xMn–xNb alloys is in the same range of that of the Ti–27Nb–xMn alloys, despite the remarkably higher amount of alloying elements of the latter. The same trends and considerations do apply to the variation of the UTS with the amount of alloying elements (Fig. 7d) and, consequently, the Ti–xMn–xNb alloys are characterised by elongation values (Fig. 7e) in between those of binary Ti–Nb and Ti–Mn alloys where local variations are, once again, primarily due to the presence of the residual porosity. Furthermore, the Ti–27Nb–xMn alloys, which are β Ti alloys, have significantly higher ductility with respect to all the other alloys, which are mainly α + β Ti alloys. Considering the variation of the hardness (Fig. 7f), the Ti–xMn–xNb alloys are still sitting in between the binary Ti–Nb and Ti–Mn alloys due to the relative hardening powder of Nb and Mn. A general increasing trend of the hardness with the amount of alloying elements is found for all the alloys, with the exception of the Ti–7Mn–xNb alloys (x = 3, 7, and 10 wt.%)^26^, where the hardness decreases as the amount of Nb increases.

Concluding, in this wok a series of Ti–xMn–xNb alloys (x = 0.5, 1, 3.5 and 5 wt.%) were developed to be processed using the blended elemental powder metallurgy approach where the powder blends are cold uniaxially pressed and sintered. The effect of the combined addition of a eutectoid (i.e. Mn) and an isomorphous (i.e. Nb) beta stabiliser on the processability, phase stability, microstructure, mechanical properties, failure mechanism, and hardening behaviour was evaluated. The incremental addition of the Mn and Nb powders decreases the compressibility of the powder blends and, consequently, the relative density decreases despite the fast diffusion of Mn which aids solid-state sintering. The Ti–xMn–xNb alloys are exclusively composed of the α-Ti and β-Ti phases and characterised by a lamellar microstructure, which is progressively refined by higher additions of Mn and Nb. Accordingly, strength and hardness increase monotonically, ductility decreases (and it is much more influenced by the residual porosity), the failure mode changes from purely intergranular to intergranular plus transgranular, and higher strain hardening decreasing rates are obtained for higher additions of the alloying elements.

## Methods

The raw elemental powders used to manufacture the Ti–xMn–xNb alloys were an irregular Ti powder (D_90_ < 75 µm, purity > 99.4%) supplied by GoodFellow, an angular Mn (D_90_ < 45 µm, purity > 99.0%) supplied by Sigma-Aldrich, and an irregular Nb powder (D_90_ < 45 µm, purity > 99.8%) supplied by AlfaAesar.

Powder blends of the Ti–xMn–xNb alloys, where x = 0.5, 1, 3.5 and 5 wt.%, were manufactured by mixing the raw powders for half an hour at a speed of 45 rpm. A servohydraulic uniaxial press was used to cold press the powder blends at 600 MPa obtaining cylindrical specimens which were subsequently consolidated at 1300 °C by means of vacuum sintering. The heating rate, soak time and cooling conditions were, respectively, 10°C/min, 2 h, and furnace cooling. The chosen pressing and sintering conditions were based on current literature about powder metallurgy Ti alloys^37–40^.

A Philips X’pert equipment with CuK_α_ radiation was used to determine the phases composing the alloys, for which X-ray patterns with 2θ = 30–80° and scanning step of 0.013° were collected. An Olympus BX53 and a Hitachi S4700 microscope was, respectively, used for optical and electron microscopy analysis of the phases of the alloys. For microstructural analysis, each material was ground up to #2000, mirror polished with colloidal SiO_2_, and eventually etched with a Kroll solution comprising distilled water, HF (2 vol.%) and HNO_3_ (4 vol.%).

A 4-digit analytical scale and a 2-digit digital gauge were used to, respectively, quantify the weight and the dimensions of the pressed samples as to be able to calculate their density. The same analytical scale was also used to perform water displacement measurements of the samples in air and in water for the calculation of the density of the sintered samples. The theoretical density of the alloys was computed considering the amount (i.e. 0.5–5 for both Mn and Nb plus Ti as balance) and the density of the elements used. For the latter, the following values were used: 4.51 g/cm^3^ for Ti, 7.43 g/cm^3^ for Mn, and 8.57 g/cm^3^ for Nb^41^.

A wire cutter was used to cut tensile test pieces with dog-bone geometry with square cross-section of 2 × 2 mm^2^ and gauge length of 20 mm. The tensile test pieces were pulled using a constant crosshead speed of 0.1 mm/min using a 33-R-204 Instron machine. A 10 mm gauge length extensometer was used to record the elongation of the tensile test pieces, testing at least five samples per alloy type. The offset method was used to determine the YS of the alloys. The A-scale Rockwell hardness was used to measure the hardness of the alloys.

## Data Availability

The datasets used and/or analysed during the current study available from the corresponding author on reasonable request.

## References

[1] Geetha M, Singh AK, Asokamani R, Gogia AK (2009). Ti based biomaterials, the ultimate choice for orthopaedic implants: A review. Prog. Mater Sci..

[2] Bolzoni L, Ruiz-Navas EM, Gordo E (2014). Investigation of the factors influencing the tensile behaviour of PM Ti-3Al-2.5V alloy. Mater. Sci. Eng. A.

[3] Yi CB, Ke ZY, Zhang L, Tan J, Jiang YH, He ZY (2020). Antibacterial Ti-Cu alloy with enhanced mechanical properties as implant applications. Mater. Res. Exp..

[4] Bolzoni L, Ruiz-Navas EM, Gordo E (2012). Influence of vacuum hot-pressing temperature on the microstructure and mechanical properties of Ti-3Al-2.5V alloy obtained by blended elemental and master alloy addition powders. Mater. Chem. Phys..

[5] Niinomi M (2008). Mechanical biocompatibilities of titanium alloys for biomedical applications. J. Mech. Behav. Biomed. Mater..

[6] Perl DP (1985). Relationship of aluminum to Alzheimer's disease. Environ. Health Persp..

[7] Domingo JL (2002). Vanadium and tungsten derivatives as antidiabetic agents: A review of their toxic effects. Biol. Trace Elem. Res..

[8] Gouda MK, Nakamura K, Gepreel MAH (2016). Effect of Mn-content on the deformation behavior of binary Ti-Mn alloys. Key Eng. Mater..

[9] Bolzoni L, Alqattan M, Peters L, Alshammari Y, Yang F (2020). Ternary Ti alloys functionalised with antibacterial activity. Sci. Rep..

[10] Cho K, Niinomi M, Nakai M, Hieda J, Fernandes Santos P, Itoh Y, Ikeda M (2014). Mechanical properties, microstructures, and biocompatibility of low-cost β-type Ti-Mn alloys for biomedical applications. Biomater. Sci. Process. Prop. Appl..

[11] Strause L, Saltman P, Glowacki J (1987). The effect of deficiencies of manganese and copper on osteoinduction and on resorption of bone particles in rats. Calcif. Tissue Int..

[12] Sigel H (2000). Metal Ions in Biological Systems: Volume 37: Manganese and Its Role in Biological Processes Metal Ions in Biological Systems.

[13] Santamaria AB (2008). Manganese exposure essentiality & toxicity. Indian J. Med. Res..

[14] Takeda S, Kakiuchi H, Doi H, Nakamura M (1989). Cytotoxicity of pure metals. Shika Zairyo Kikai.

[15] Fernandes Santos P, Niinomi M, Liu H, Cho K, Nakai M, Itoh Y, Narushima T, Ikeda M (2016). Fabrication of low-cost beta-type Ti-Mn alloys for biomedical applications by metal injection molding process and their mechanical properties. J. Mech. Behav. Biomed. Mater..

[16] Kim J-W, Hwang M-J, Han M-K, Kim Y-G, Song H-J, Park Y-J (2016). Effect of manganese on the microstructure, mechanical properties and corrosion behavior of titanium alloys. Mater. Chem. Phys..

[17] Han M-K, Kim J-Y, Hwang M-J, Song H-J, Park Y-J (2015). Effect of Nb on the microstructure mechanical properties, corrosion behavior, and cytotoxicity of Ti-Nb alloys. Materials.

[18] Lee CM, Ju CP, Chern Lin JH (2002). Structure-property relationship of cast Ti-Nb alloys. J. Oral Rehab..

[19] Xu L-J, Xiao S-L, Tian J, Chen Y-Y, Huang Y-D (2009). Microstructure and dry wear properties of Ti-Nb alloys for dental prostheses. Trans. Nonferrous Metals Soc. China.

[20] Kikuchi M, Takahashi M, Okuno O (2003). Mechanical properties and grindability of dental cast Ti-Nb alloys. Dent. Mater. J..

[21] Zhang Y, Sun D, Cheng J, Tsoi JKH, Chen J (2020). Mechanical and biological properties of Ti-(0–25 wt%)Nb alloys for biomedical implants application. Regen. Biomater..

[22] Zhao D, Chang K, Ebel T, Qian M, Willumeit R, Yan M, Pyczak F (2013). Microstructure and mechanical behavior of metal injection molded Ti-Nb binary alloys as biomedical material. J. Mech. Behav. Biomed. Mater..

[23] Yılmaz E, Gökçe A, Findik F, Gulsoy HO (2018). Metallurgical properties and biomimetic HA deposition performance of Ti-Nb PIM alloys. J. Alloy. Compd..

[24] Kalita D, Rogal Ł, Czeppe T, Wójcik A, Kolano-Burian A, Zackiewicz P, Kania B, Dutkiewicz J (2020). Microstructure and mechanical properties of Ti-Nb alloys prepared by mechanical alloying and spark plasma sintering. J. Mater. Eng. Perform..

[25] Chen Z, Liu Y, Jiang H, Liu M, Wang CH, Cao GH (2017). Microstructures and mechanical properties of Mn modified, Ti-Nb-based alloys. J. Alloy. Compd..

[26] Ehtemam-Haghighi S, Attar H, Dargusch MS, Kent D (2019). Microstructure, phase composition and mechanical properties of new, low cost Ti-Mn-Nb alloys for biomedical applications. J. Alloy. Compd..

[27] Raynova S, Collas Y, Yang F, Bolzoni L (2019). Advancement in the pressureless sintering of CP titanium using high-frequency induction heating. Metall. Mater. Trans. A..

[28] Amherd Hidalgo A, Frykholm R, Ebel T, Pyczak F (2017). Powder metallurgy strategies to improve properties and processing of titanium alloys: A review. Adv. Eng. Mater..

[29] Bolzoni L, Paul M, Yang F (2022). Effect of combined lean additions of isomorphous and eutectoid beta stabilisers on the properties of titanium. J. Market. Res..

[30] Wang Q, Dong C, Liaw PK (2015). Structural stabilities of β-Ti alloys studied using a new mo equivalent derived from [β/(α + β)] phase-boundary slopes. Metall. Mater. Trans. A..

[31] Kumar P, Chandran KSR (2017). Strength-ductility property maps of powder metallurgy (PM) Ti-6Al-4V alloy: A critical review of processing-structure-property relationships. Metall. Mater. Trans. A..

[32] Wang H, Fang ZZ, Sun P (2010). A critical review of mechanical properties of powder metallurgy titanium. Int. J. Powder Metall..

[33] Kocks UF, Mecking H (2003). Physics and phenomenology of strain hardening: The FCC case. Prog. Mater Sci..

[34] Yasnikov IS, Vinogradov A, Estrin Y (2014). Revisiting the considère criterion from the viewpoint of dislocation theory fundamentals. Scripta Mater..

[35] Alshammari Y, Jia M, Yang F, Bolzoni L (2020). The effect of α+ β forging on the mechanical properties and microstructure of binary titanium alloys produced via a cost-effective powder metallurgy route. Mater. Sci. Eng., A.

[36] Cho K, Niinomi M, Nakai M, Liu H, Santos PF, Itoh Y, Ikeda M, Gepreel MA-H, Narushima T (2016). Improvement in mechanical strength of low-cost β-type Ti–Mn alloys fabricated by metal injection molding through cold rolling. J. Alloy. Compd..

[37] Raynova S, Imam MA, Yang F, Bolzoni L (2019). Hybrid microwave sintering of blended elemental Ti alloys. J. Manuf. Process..

[38] Sjafrizal T, Dehghan-Manshadi A, Kent D, Yan M, Dargusch MS (2020). Effect of Fe addition on properties of Ti-6Al-xFe manufactured by blended elemental process. J. Mech. Behav. Biomed. Mater..

[39] Jia MT, Gabbitas B, Bolzoni L (2018). Evaluation of reactive induction sintering as a manufacturing route for blended elemental Ti-5Al-2.5Fe alloy. J. Mater. Process. Technol..

[40] Zhang E, Wang X, Chen M, Hou B (2016). Effect of the existing form of Cu element on the mechanical properties, bio-corrosion and antibacterial properties of Ti-Cu alloys for biomedical application. Mater. Sci. Eng., C.

[41] American Society for Metals - ASM, ASM Handbook Volume 2: Properties and Selection: Nonferrous Alloys and Special-purpose Materials, Ohio, USA, 1990.

